# Signal parameter estimation using fourth order statistics: multiplicative and additive noise environment

**DOI:** 10.1186/s40064-015-1085-5

**Published:** 2015-06-24

**Authors:** Chandrakant J Gaikwad, Hemant K Samdani, Pradip Sircar

**Affiliations:** Department of Electrical Engineering, Indian Institute of Technology Kanpur, Kanpur, UP 208016 India

**Keywords:** Parameter estimation, Multiplicative noise, Fourth-order cumulant, Higher-order statistics

## Abstract

Parameter estimation of various multi-component stationary and non-stationary signals in multiplicative and additive noise is considered in this paper. It is demonstrated that the parameters of complex sinusoidal signal, complex frequency modulated (FM) sinusoidal signal and complex linear chirp signal in presence of additive and multiplicative noise can be estimated using a new definition of the fourth order cumulant (FOC), and the computed accumulated FOC (AFOC). Analytical expressions for the FOC/AFOC of the above signals are derived. The concept of accumulated cumulant is introduced to handle the case of a non-stationary signal, for which the fourth order cumulant may be a function of both time and lag. Simulation study is carried out for all the three signals. In case of complex sinusoidal signals, the resul ts of parameter estimation show that the proposed method based on the new definition of fourth order cumulant performs better than an existing method based on fourth order statistics. The proposed method can be employed for parameter estimation of non-stationary signals also as mentioned above. For comparison purpose, the Cramer-Rao (CR) bound expressions are derived for all the signals considered for parameter estimation. The simulation results for non-stationary signals are compared with the CR bounds.

## Background

In many applications, such as Doppler radar signal processing (Besson and Castanie 1993), synthetic aperture radar image processing (Frost et al. 1982; Lee and Jurkevich 1994), optical imaging under speckle or scintillation condition (Frankot and Chellappa 1987; Jain 2002), transmission of signals over fading channels (Makrakis and Mathiopoulos 1990a, b; Proakis 2001), speech processing in signal-dependent noise (Kajita and Itakura 1995; Quatieri 2002), and more, we need to consider the noise component to be both multiplicative and additive to the signal component.

In literature, signal parameter estimation in multiplicative and additive noise has been reported employing the non-linear least squares (NLLS) techniques (Besson and Stoica 1995; Besson and Stoica 1998; Ghogho et al. 2001; Besson et al. 1999), the cyclostationary approaches (Shamsunder et al. 1995; Zhou and Giannakis 1995; Giannakis and Zhou 1995; Ghogho et al. 1999a, 1999b), and the methods based on higher order statistics (Dwyer 1991; Swami 1994; Zhou and Giannakis 1994). In the NLLS techniques, a random amplitude observed signal is matched with a constant amplitude modelled signal in the least squares sense. When the random amplitude process is zero mean, we match the squared observed signal with the squared modelled signal. The NLLS estimators lead to an optimization problem which needs to be solved by an iterative technique. For a linear chirp signal, we need to perform a two-dimensional search where the initial guess, global convergence, convergence rate, and more are crucial issues (Besson et al. 1999). In the approaches based on cyclic statistics, we utilize the properties of the underlying signal. For a random amplitude polynomial phase signal, if the polynomial order is $(p+1)$, then the process will be $2^p$-order cyclostationary, i.e., the signal moments and cumulants of order $2^p$ will be (almost) periodic. Using the cyclic moments/cumulants of order $2^p$, the $(p+1)$th order coefficient in the phase polynomial can be estimated. Having estimated the highest order polynomial coefficient, the signal can be demodulated to reduce the polynomial order, and the process can be repeated to estimate the next highest order polynomial coefficient. For the cyclic estimator to work, it is necessary that the random amplitude process be bandlimited, and higher the polynomial order, the more stringent the requirement on the bandlimitedness of the amplitude process. Some other issues are: (1) When finite data samples are used, the peaks in the cyclic moments/cumulants may be difficult to discern; (2) Due to the sequential procedure, there is cumulative effect that significantly degrades the accuracy of lower order polynomial coefficients (Shamsunder et al. 1995).

In the present work, our focus is on higher order statistics. We do not consider any other approaches for comparison or otherwise. In the methods based on higher order statistics, our concern is to develop a way to reduce the higher dimensionality of higher order moments and cumulants. Another issue is to tackle the non-stationarity of the observed signal, which makes the moments and cumulants time-varying in nature. In the paper, we address these issues and find some solutions.

It is known that the cumulants of order greater than two of Gaussian processes are zero, whereas the cumulants of non-Gaussian processes carry higher order statistical information. Therefore, when the additive noise process is Gaussian and the signal process modulated by the multiplicative noise is non-Gaussian, one may use the methods based on third or fourth order cumulants of the signal for estimating signal parameters (Swami and Mendel 1991; Swami 1994).

Different slices of higher order cumulants are utilized for parameter estimation of various harmonic and modulated signals. Higher dimensionality of higher order cumulants are conventionally tackled by taking appropriate slices of cumulants such that the slices retain the pertinent information about the signal (Swami and Mendel 1991; Swami 1994). However, the selection of appropriate slices for various signals of interest may be a complicated task. Moreover, when the signal is non-stationary in nature, the moments and cumulants of the signal may depend on both time and lag (Sircar and Mukhopadhyay 1995; Sircar and Syali 1996; Sircar and Sharma 1997; Sircar and Saini 2007). Therefore, the utilization of such time-varying moments and cumulants for parameter estimation of signals may be quite challenging.

In the accompanying paper, a new definition for calculating the symmetric fourth order moment and cumulant of a transient signal has been proposed (Sircar et al. 2015). It has been demonstrated that with the choice of the lag-parameters in the definition, the computed moment and cumulant of the non-stationary signal will have some desirable properties. In the present work, we use the same definition for computing the symmetric fourth order moments and cumulants of some stationary and non-stationary signals in multiplicative and additive noise.

The multi-component signals considered in this paper for parameter estimation are complex sinusoidal signal, complex frequency modulated (FM) sinusoidal signal, and complex linear chirp signal. The complex amplitude modulated (AM) sinusoidal signal case can be treated as an extension of the complex sinusoidal signal case with main and side lobes. Thus, this case is not considered separately. The concept of accumulated fourth order moment, as developed in the accompanying paper (Sircar et al. 2015), has been extended to the concept of accumulated fourth order cumulant while estimating parameters of the complex FM sinusoidal signal in multiplicative noise.

The paper is organized as follows: In "Symmetric fourth order cumulant", we give the definition of fourth order moment and cumulant used in this work, and derive the analytical expressions for the symmetric fourth order cumulant or accumulated cumulant of the above multi-component signals in multiplicative and additive noise. We analyze the "Deterministic signal case" and discuss the effects of replacing the ensemble average by the time average. In the next section "Simulation study" is presented, and the "Conclusion" is given in last section. The Cramer-Rao (CR) bound expressions for the simulated examples are derived in Appendices A–C.

## Symmetric fourth order cumulant

Consider the complex-valued discrete-time signal *Y*[*n*] comprising of the sum of *M* signals in presence of multiplicative and additive noise,

1 $$\begin{aligned} Y[n]&= \sum \limits _{i=1}^{M}A_{i}[n]S_{i}[n]+W[n]\\ \nonumber&=X[n]+W[n] \end{aligned}$$

where $A_{i}[n]$ is the *i*th multiplicative noise process, $S_{i}[n]$ is the *i*th signal process, *W*[*n*] is the additive noise process, and *X*[*n*] is the composite signal component comprising of multi-component signal and multiplicative noise.

It is assumed that *W*[*n*] is the zero-mean complex Gaussian noise process independent of the multiplicative noise processes. Since the fourth order moment and cumulant of the Gaussian process are zero, we need to study the fourth order statistics of *X*[*n*], which will be same as that of *Y*[*n*].

We define the symmetric fourth order moment (FOM) $R_{4X} [n,k]$ of the sequence *X*[*n*] as follows (Sircar et al. 2015),

2 $$\begin{aligned} R_{4X} [n,k] = \mathcal{{E}} \left\{ X^{\star }[n] X[n+k] X^{\star }[-n] X[-n+k] \right\} \end{aligned}$$

where $\mathcal{{E}}$ is the expectation operator and $^\star$ denotes complex conjugation.

The symmetric fourth-order cumulant of *X*[*n*] is defined as

3 $$\begin{aligned} C_{4X}[n,k]&=\mathcal{{E}}\left\{ X^{\star }[n] X[n+k] X^{\star }[-n] X[-n+k] \right\} \nonumber \\ &\quad-\, \mathcal{{E}}\left\{ X^{\star }[n] X[n+k] \right\} \mathcal{{E}}\left\{ X^{\star }[-n] X[-n+k] \right\} \nonumber \\ &\quad-\, \mathcal{{E}}\left\{ X^{\star }[n] X^{\star }[-n] \right\} \mathcal{{E}}\left\{ X[n+k] X[-n+k] \right\} \nonumber \\&\quad -\, \mathcal{{E}}\left\{ X^{\star }[n] X[-n+k] \right\} \mathcal{{E}}\left\{ X^{\star }[-n] X[n+k] \right\} \end{aligned}$$

We will compute the symmetric fourth order cumulants of different signal models considered in the sequel, and if the fourth order cumulant is a function of both time *n* and lag *k*, we will use the concept of accumulated fourth order cumulant (AFOC) (Sircar and Mukhopadhyay 1995; Sircar et al. 2015). The resulting AFOC sequence will be a function of lag only.

### Complex sinusoidal signals

The discrete-time signal *X*[*n*] consisting of *M* complex sinusoids of angular frequencies $\omega _i$’s in multiplicative noise can be expressed as

4 $$\begin{aligned} X[n]=\sum _{i=1}^M \alpha _i e^{j\left( \omega _i n+ \phi _i\right) } \end{aligned}$$

where $\alpha _i$’s are assumed to be independent and identically distributed (i.i.d.) random variables, and $\phi _i$’s are assumed to be i.i.d. and $U[0,2\pi )$.

By using the definition of the FOM $R_{4X}[n,k]$ of *X*[*n*] as given by (2), we compute

5 $$\begin{aligned} R_{4X}[n,k]&=\mathcal{{E}}\Bigg \{\sum _{i=1}^M \alpha _i e^{-j\left( \omega _i n+ \phi _i\right) } \sum _{u=1}^M \alpha _u e^{j\left[ \omega _u(n+k)+\phi _u\right] } \nonumber \\&\quad \times \sum _{l=1}^M \alpha _l e^{-j\left( -\omega _l n+ \phi _l\right) } \sum _{v=1}^M \alpha _v e^{j\left[ \omega _v(-n+k)+\phi _v\right] } \Bigg \} \nonumber \\&= \sum _{u} \sum _{v} \mathcal{{E}}\big \{\alpha _u^2\big \}\mathcal{{E}}\big \{\alpha _v^2\big \} e^{j(\omega _u+\omega _v)k} \nonumber \\ & \quad + \sum _{u} \sum _{v} \mathcal{{E}}\big \{\alpha _u^2\big \}\mathcal{{E}}\big \{\alpha _v^2\big \} e^{j2(\omega _u-\omega _v)n} e^{j(\omega _u+\omega _v)k} \nonumber \\ & \quad - \sum _{u} \mathcal{{E}}\big \{\alpha _u^4\big \} e^{j2\omega _uk} \end{aligned}$$

where the following results of expectation are used:

6 $$\begin{aligned} \mathcal{{E}} \left\{ e^{j(-\phi _i + \phi _u - \phi _l + \phi _v)} \right\}&=1 \quad \text{ when } i=u\text{, } l=v\text{, } \text{ and } u{\ne }v \nonumber \\&=1 \quad \text{ when } i=v\text{, } l=u\text{, } \text{ and } u{\ne }v \nonumber \\&= 1 \quad \text{ when } i=u=l=v \nonumber \\&= 0 \quad \text{ otherwise } \end{aligned}$$

Note that in (5), the third case ($i = u = l = v$) is added twice in the first two summations and subtracted once in the last summation, which leaves an overall inclusion of one term of this case.

On further simplification of (5), we get

7 $$\begin{aligned} R_{4X}[n,k]= & {} \sum _{u} \sum _{v} r_{2\alpha }^2 e^{j(\omega _u+\omega _v)k} + \sum _{u} \sum _{v} r_{2\alpha }^2 e^{j2(\omega _u-\omega _v)n} e^{j(\omega _u+\omega _v)k} \nonumber \\ & {}- \sum _{u} r_{4\alpha } e^{j2\omega _uk} \end{aligned}$$

where $r_{2\alpha }=\mathcal{{E}}\left\{ \alpha ^2\right\}$ and $r_{4\alpha }=\mathcal{{E}}\left\{ \alpha ^4\right\}$ are the second and fourth order moments, respectively, of $\alpha _i$’s.

We now compute the fourth-order cumulant $C_{4X}[n,k]$ of *X*[*n*] as defined by (3),

8 $$\begin{aligned} C_{4X}[n,k]&= R_{4X}[n,k]-\mathcal{{E}}\left\{ \sum _{i=1}^M \alpha _i e^{-j\left( \omega _i n+ \phi _i\right) } \sum _{u=1}^M \alpha _u e^{j\left[ \omega _u(n+k)+\phi _u\right] }\right\} \nonumber \\&\quad \times \mathcal{{E}}\left\{ \sum _{l=1}^M \alpha _l e^{-j\left( -\omega _l n+ \phi _l\right) } \sum _{v=1}^M \alpha _v e^{j\left[ \omega _v(-n+k)+\phi _v\right] } \right\} \nonumber \\&\quad -\,\mathcal{{E}}\left\{ \sum _{i=1}^M \alpha _i e^{-j\left( \omega _i n+ \phi _i\right) } \sum _{l=1}^M \alpha _l e^{-j\left( -\omega _l n+ \phi _l\right) } \right\} \nonumber \\&\quad \times\mathcal{{E}}\left\{ \sum _{u=1}^M \alpha _u e^{j\left[ \omega _u(n+k)+\phi _u\right] } \sum _{v=1}^M \alpha _v e^{j\left[ \omega _v(-n+k)+\phi _v\right] } \right\} \nonumber \\&\quad-\,\mathcal{{E}}\left\{ \sum _{i=1}^M \alpha _i e^{-j\left( \omega _i n+ \phi _i\right) } \sum _{v=1}^M \alpha _v e^{j\left[ \omega _v(-n+k)+\phi _v\right] } \right\} \nonumber \\&\quad\times\mathcal{{E}}\left\{ \sum _{l=1}^M \alpha _l e^{-j\left( -\omega _l n+ \phi _l\right) }\sum _{u=1}^M \alpha _u e^{j\left[ \omega _u(n+k)+\phi _u\right] }\right\} \end{aligned}$$

Note that the first term $R_{4X}[n,k]$ of (8) has already been computed, and

9 $$\begin{aligned} \text{ the } \text{ second } \text{ term }=-\sum _{u} \sum _{v} r_{2\alpha }^2 e^{j(\omega _u+\omega _v)k} \end{aligned}$$

where we use the expectation

10 $$\begin{aligned} \mathcal{{E}} \left\{ e^{j(-\phi _i + \phi _u)} \right\}&=1 \quad \text{ when }\, i=u \nonumber \\&=0 \quad \text{ otherwise } \end{aligned}$$

Moreover, the third term of (8) is found to be identically zero, and

11 $$\begin{aligned} \text{ the } \text{ fourth } \text{ term }=-\sum _{u} \sum _{v} r_{2\alpha }^2 e^{j2(\omega _u-\omega _v)n} e^{j(\omega _u+\omega _v)k} \end{aligned}$$

where again we use the expectations (10) and

12 $$\begin{aligned} \mathcal{{E}} \left\{ e^{j\phi _i} \right\} = 0 \end{aligned}$$

Substituting the evaluated results of all terms in (8), we get

13 $$\begin{aligned} C_{4X}[k]=-\sum _{u} r_{4\alpha } e^{j2\omega _uk} \end{aligned}$$

Note that the fourth-order cumulant $C_{4X}$ is time-invariant as expected, because the signal *X*[*n*] of (4) is a stationary signal. Once the FOC sequence is computed, it is easy to extract its frequencies which are set at twice the frequencies of the signal.

### Complex FM sinusoidal signals

The discrete-time signal *X*[*n*] consisting of *M* complex frequency modulated (FM) sinusoids of carrier angular frequencies $\omega _i$’s, modulating angular frequencies $\xi _i$’s and modulation indices $\beta _i$’s in multiplicative noise can be expressed as

14 $$\begin{aligned} X[n]=\sum _{i=1}^M \alpha _i e^{j\left[ \omega _i n+\beta _i \sin \left( \xi _in\right) +\phi _i\right] } \end{aligned}$$

where we assume that $\alpha _i$’s are i.i.d. random variables, and $\phi _i$’s are i.i.d. and $U[0,2\pi )$

By using the notation

15 $$\begin{aligned} \rho _{i,n}=\omega _in+\beta _i \sin \left( \xi _in\right) \end{aligned}$$

and the definition of the fourth-order moment $R_{4X}[n,k]$ of *X*[*n*] as given by (2), we calculate

16 $$\begin{aligned} R_{4X}[n,k]&= \mathcal{{E}}\left\{ \sum _{i=1}^M \alpha _i e^{-j\left( \rho _{i,n}+\phi _i\right) } \sum _{u=1}^M \alpha _u e^{j\left( \rho _{u,n+k}+\phi _u\right) } \right. \nonumber \\&\quad\times\left. \sum _{l=1}^M \alpha _l e^{-j\left( \rho _{l,-n}+\phi _l\right) } \sum _{v=1}^M \alpha _v e^{j\left( \rho _{v,-n+k}+\phi _v\right) } \right\} \nonumber \\&= \sum _{u}\sum _{v}r_{2\alpha }^2 e^{j\left( -\rho _{u,n}+\rho _{u,n+k}-\rho _{v,-n}+\rho _{v,-n+k}\right) } \nonumber \\&\quad+\sum _{u}\sum _{v}r_{2\alpha }^2 e^{j\left( -\rho _{v,n}+\rho _{v,-n+k}-\rho _{u,-n}+\rho _{u,n+k}\right) } \nonumber \\&\quad-\sum _{u}r_{4\alpha } e^{j\left( -\rho _{u,n}+\rho _{u,n+k}-\rho _{u,-n}+\rho _{u,-n+k}\right) } \end{aligned}$$

where the expectations (6) are used, and the second and fourth order moments of $\alpha _i$’s are substituted.

Next, we compute the fourth-order cumulant $C_{4X}[n,k]$ of *X*[*n*] as given by (3),

17 $$\begin{aligned} C_{4X}[n,k]&= R_{4X}[n,k]-\mathcal{{E}}\left\{ \sum _{i=1}^M \alpha _i e^{-j\left( \rho _{i,n}+\phi _i\right) } \sum _{u=1}^M \alpha _u e^{j\left( \rho _{u,n+k}+\phi _u\right) }\right\} \nonumber \\&\quad \times \mathcal{{E}}\left\{ \sum _{l=1}^M \alpha _l e^{-j\left( \rho _{l,-n}+\phi _l\right) } \sum _{v=1}^M \alpha _v e^{j\left( \rho _{v,-n+k}+\phi _v\right) } \right\} \nonumber \\&\quad-\mathcal{{E}}\left\{ \sum _{i=1}^M \alpha _i e^{-j\left( \rho _{i,n}+\phi _i\right) } \sum _{l=1}^M \alpha _l e^{-j\left( \rho _{l,-n}+\phi _l\right) }\right\} \nonumber \\&\quad \times \mathcal{{E}}\left\{ \sum _{u=1}^M \alpha _u e^{j\left( \rho _{u,n+k}+\phi _u\right) } \sum _{v=1}^M \alpha _v e^{j\left( \rho _{v,-n+k}+\phi _v\right) } \right\} \nonumber \\&\quad - \mathcal{{E}}\left\{ \sum _{i=1}^M \alpha _i e^{-j\left( \rho _{i,n}+\phi _i\right) } \sum _{v=1}^M \alpha _v e^{j\left( \rho _{v,-n+k}+\phi _v\right) }\right\} \nonumber \\&\quad \times \mathcal{{E}}\left\{ \sum _{l=1}^M \alpha _l e^{-j\left( \rho _{l,-n}+\phi _l\right) }\sum _{u=1}^M \alpha _u e^{j\left( \rho _{u,n+k}+\phi _u\right) }\right\} \end{aligned}$$

The first term $R_{4X}[n,k]$ of (17) has been computed, and

18 $$\begin{aligned} \text{ the } \text{ second } \text{ term }=-\sum _{u}\sum _{v} r_{2\alpha }^2 e^{j\left( -\rho _{u,n}+\rho _{u,n+k}-\rho _{v,-n}+\rho _{v,-n+k}\right) } \end{aligned}$$

where the expectation (10) is used. The third term of (17) is identically zero, and

19 $$\begin{aligned} \text{ the } \text{ fourth } \text{ term }=-\sum _{u}\sum _{v} r_{2\alpha }^2 e^{j\left( -\rho _{v,n}+\rho _{v,-n+k}-\rho _{u,-n}+\rho _{u,n+k}\right) } \end{aligned}$$

where the expectations (10) and (12) are used.

Substituting all terms in (17), we find

20 $$\begin{aligned} C_{4X}[n,k]=-\sum _{u} r_{4\alpha } e^{j\left( -\rho _{u,n}+\rho _{u,n+k}-\rho _{u,-n}+\rho _{u,-n+k}\right) } \end{aligned}$$

and using (15) for $\rho _{u,n}$’s, we get after simplification

21 $$\begin{aligned} C_{4X}[n,k]=-\sum _{u} r_{4\alpha } e^{j\left[ 2\omega _u k+2\beta _u\cos \left( \xi _u n\right) \sin \left( \xi _u k\right) \right] } \end{aligned}$$

which can be further simplified to yield

22 $$\begin{aligned} C_{4X}[n,k]&= -\sum _{u} r_{4\alpha } e^{j2\omega _u k}-\sum _{u}r_{4\alpha }\beta _u\cos \left( \xi _u n\right) e^{j\left( 2\omega _u+\xi _u\right) k}\nonumber \\&\quad+ \sum _{u} r_{4\alpha }\beta _u\cos \left( \xi _u n\right) e^{j\left( 2\omega _u-\xi _u\right) k} \end{aligned}$$

under the assumption that the signal *X*[*n*] comprises of narrow-band FM sinusoids with small values of $\beta _u$’s.

Note that the FOC $C_{4X}[n,k]$ is now a function of both time *n* and lag *k*. This is not unexpected because the signal *X*[*n*] of (14) is a non-stationary signal (Sircar and Sharma 1997; Sircar and Saini 2007). We compute the accumulated FOC (AFOC) $Q_{4X}$ by summing $C_{4X}$ over an appropriately selected time frame $[n_1,n_2]$ (Sircar and Mukhopadhyay 1995; Sircar et al. 2015),

23 $$\begin{aligned} Q_{4X}[k]= & {} \sum _{n=n_1}^{n_2}C_{4X}[n,k]\nonumber \\= & {} \sum _{u}E e^{j2\omega _uk}+\sum _{u}F_u e^{j\left( 2\omega _u+\xi _u\right) k} -\sum _{u}F_u e^{\left( 2\omega _u-\xi _u\right) k} \end{aligned}$$

where $$E=-r_{4\alpha }\left( n_2-n_1+1\right)$$ and $$F_u=-r_{4\alpha }\beta _u\sum _{n=n_1}^{n_2}\cos \left( \xi _un\right)$$.

Once the AFOC sequence is computed, we extract its frequencies which are set at twice the carrier frequencies of the signal *X*[*n*], together with the side-frequencies at 2 times carrier plus/minus modulating frequencies.

### Complex linear chirp signals

The discrete time signal *X*[*n*] consisting of *M* complex linear chirps of on-set angular frequencies $\omega _i$’s and rates of increase of angular frequencies or chirp rates $\gamma _i$’s in multiplicative noise can be expressed as

24 $$\begin{aligned} X[n]=\sum _{i=1}^M \alpha _{i} e^{j(\omega _{i}n+\gamma _{i}n^{2}/2+ \phi _{i})} \end{aligned}$$

where $\alpha _i$’s are assumed to be i.i.d random variables, and $\phi _i$’s are assumed to be i.i.d and $U[0,2\pi )$. The fourth-order moment $R_{4X}[n,k]$ of *X*[*n*] is computed by (2) as follows

25 $$\begin{aligned}R_{4X}[n,k]&=\mathcal{{E}}\Bigg \{\sum _{i=1}^M \alpha _i e^{-j\left( \omega _{i}n+\gamma _{i}n^{2}/2+ \phi _{i}\right) } \sum _{u=1}^M \alpha _u e^{j\left[ \omega _u(n+k)+\gamma _{u}(n+k)^{2}/2 +\phi _u\right] } \nonumber \\&\quad\times\sum _{l=1}^M \alpha _l e^{-j\left( -\omega _l n+\gamma _{l}n^{2}/2 +\phi _l\right) } \sum _{v=1}^M \alpha _v e^{j\left[ \omega _v(-n+k)+\gamma _{v}(-n+k)^{2}/2 +\phi _v\right] } \Bigg \} \nonumber \\&= \sum _{u} \sum _{v} r_{2\alpha }^2 e^{j[(\omega _u+\omega _v)k+(\gamma _{u}+\gamma _{v})k^2/2]} \nonumber \\& \quad + \sum _{u} \sum _{v} r_{2\alpha }^2 e^{j[2(\omega _u-\omega _v)n+(\gamma _{u}-\gamma _{v})nk]}e^{j[(\omega _u+\omega _v)k+(\gamma _{u}+\gamma _{v})k^2/2]} \nonumber \\&\quad- \sum _{u} r_{4\alpha } e^{j(2\omega _uk+\gamma _uk^2)} \end{aligned}$$

where we use the expectations (6) and substitute the second and fourth order moments of $\alpha _i$’s.

The fourth-order cumulant $C_{4X}[n,k]$ of *X*[*n*] as given by (3), is computed as

26 $$\begin{aligned}C_{4X}[n,k]&=R_{4X}[n,k]\nonumber \\&\quad-\mathcal{{E}}\left\{ \sum _{i=1}^M \alpha _i e^{-j\left( \omega _{i}n+\gamma _{i}n^{2}/2+ \phi _i\right) } \sum _{u=1}^M \alpha _u e^{j\left[ \omega _u(n+k)+\gamma _{u}(n+k)^{2}/2+\phi _u\right] }\right\} \nonumber \\&\quad\times\mathcal{{E}}\left\{ \sum _{l=1}^M \alpha _l e^{-j\left( -\omega _l n+\gamma _{l}n^{2}/2+ \phi _l\right) } \sum _{v=1}^M \alpha _v e^{j\left[ \omega _v(-n+k)+\gamma _{v}(-n+k)^{2}/2+\phi _v\right] } \right\} \nonumber \\&\quad-\mathcal{{E}}\left\{ \sum _{i=1}^M \alpha _i e^{-j\left( \omega _{i}n+\gamma _{i}n^{2}/2+ \phi _i\right) } \sum _{l=1}^M \alpha _l e^{-j\left( -\omega _l n+\gamma _{l}n^{2}/2+ \phi _l\right) } \right\} \nonumber \\&\quad \times \mathcal{{E}}\left\{ \sum _{u=1}^M \alpha _u e^{j\left[ \omega _u(n+k)+\gamma _{u}(n+k)^{2}/2+\phi _u\right] } \sum _{v=1}^M \alpha _v e^{j\left[ \omega _v(-n+k)+\gamma _{v}(-n+k)^{2}/2+\phi _v\right] } \right\} \nonumber \\&\quad-\mathcal{{E}}\left\{ \sum _{i=1}^M \alpha _i e^{-j\left( \omega _{i}n+\gamma _{i}n^{2}/2+ \phi _i\right) } \sum _{v=1}^M \alpha _v e^{j\left[ \omega _v(-n+k)+\gamma _{v}(-n+k)^{2}/2+\phi _v\right] } \right\} \nonumber \\&\quad \times \mathcal{{E}}\left\{ \sum _{l=1}^M \alpha _l e^{-j\left( -\omega _l n+\gamma _{l}n^{2}/2+ \phi _l\right) }\sum _{u=1}^M \alpha _u e^{j\left[ \omega _u(n+k)+\gamma _{u}(n+k)^{2}/2+\phi _u\right] }\right\} \end{aligned}$$

The first term $R_{4X}[n,k]$ of (26) has already been computed, and

27 $$\begin{aligned} \text{ the } \text{ second } \text{ term }=-\sum _{u} \sum _{v} r_{2\alpha }^2 e^{j[(\omega _u+\omega _v)k+(\gamma _{u}+\gamma _{v})k^2/2]} \end{aligned}$$

where the expectation (10) is used. The third term of (26) is identically zero, and

28 $$\begin{aligned} \text{ the } \text{ fourth } \text{ term }=-\sum _{u} \sum _{v} r_{2\alpha }^2e^{j[2(\omega _u-\omega _v)n+(\gamma _{u}-\gamma _{v})nk]}e^{j[(\omega _u+\omega _v)k+(\gamma _{u}+\gamma _{v})k^2/2]} \end{aligned}$$

where the expectations (10) and (12) are used. Substituting all the terms in (26), we get

29 $$\begin{aligned} C_{4X}[k]=-\sum _{u} r_{4\alpha } e^{j(2\omega _uk+\gamma _{u}k^{2})} \end{aligned}$$

This result is remarkable, because it shows that the symmetric FOC sequence is time-invariant. Note that the chirp signal of (24) is a non-stationary signal. However, for the choice of arguments proposed in this paper, the symmetric FOC sequence depends only on time lag and not on absolute time.

## Deterministic signal case

In this section, we discuss the non-random signal case. Although the observed sequence can be thought of as a sample of some discrete-time random process, any replacement of ensemble average by temporal average will not likely produce the same result when the underlying signal may not necessarily be stationary and ergodic.

Given a finite length sequence *X*[*n*], we compute the $\tilde{C}$-sequence as follows (Sircar et al. (2015))

30 $$\begin{aligned}\tilde{C}[k]&=\frac{1}{n_2-n_1+1} \sum _{n=n_1}^{n_2} \bar{X}^{\star }[n] \bar{X}[n+k]\bar{X}^{\star }[-n] \bar{X}[-n+k]\nonumber \\ &\quad - \,\frac{1}{(n_2-n_1+1)^2} \sum _{n=n_1}^{n_2} \bar{X}^{\star }[n] \bar{X}[n+k] \sum _{m=n_1}^{n_2} \bar{X}^{\star }[-m] \bar{X}[-m+k] \nonumber \\ &\quad - \frac{1}{(n_2-n_1+1)^2}\sum _{n=n_1}^{n_2} \bar{X}^{\star }[n] \bar{X}^{\star }[-n] \sum _{m=n_1}^{n_2} \bar{X}[m+k] \bar{X}[-m+k] \nonumber \\ &\quad - \,\frac{1}{(n_2-n_1+1)^2}\sum _{n=n_1}^{n_2} \bar{X}^{\star }[n] \bar{X}[-n+k] \sum _{m=n_1}^{n_2}\bar{X}^{\star }[-m] \bar{X}[m+k] \end{aligned}$$

where $\bar{X}[n]=X[n]-X_0$, $X_0$ being the mean of the finite-length data record. We call $\tilde{C}[k]$ as the fourth order time cumulant (FOTC). The choice of $n_1$ and $n_2$ should be such that there is no running off the ends of the data record (Sircar and Mukhopadhyay 1995; Sircar et al. 2015). We now compute the $\tilde{C}$-sequence for the complex sinusoidal signal. On substitution of (4) and simplification, the terms of (30) reduce to the general form as shown below:

31 $$\begin{aligned}\frac{1}{n_2-n_1+1} \sum _{n=n_1}^{n_2} \bar{X}^{\star }[n] \bar{X}[n+k]\bar{X}^{\star }[-n] \bar{X}[-n+k] &=\sum _{u=1}^M \sum _{v=1}^M t_{11}[u,v] e^{j(\omega _u+\omega _v)k} \nonumber\\&+ \sum _{u=1}^M t_{12}[u] e^{j(\omega _uk)} + t_{13}\,; \nonumber \\ - \frac{1}{(n_2-n_1+1)^2} \sum _{n=n_1}^{n_2} \bar{X}^{\star }[n] \bar{X}[n+k] \sum _{m=n_1}^{n_2} \bar{X}^{\star }[-m] \bar{X}[-m+k] &=- \sum _{u=1}^M \sum _{v=1}^M t_{21}[u,v] e^{j(\omega _u+\omega _v)k} \nonumber\\&- \sum _{u=1}^M t_{22}[u] e^{j(\omega _uk)} - t_{23}\,; \nonumber \\&\vdots \end{aligned}$$

where each coefficient $t_{\ell 1}$ is made independent of time *n* (and *m*), indices *i* and *l* (see 8) by taking summation over respective variables. Similarly, each of $t_{\ell 2}$ is independent of all variables except *u*, and every $t_{\ell 3}$ is made independent of all six variables by summation. Note that if the mean $X_0 = 0$, the coefficients $t_{\ell 2}$ and $t_{\ell 3}$ will be identically zero. In this case, each of $t_{\ell 1}$ will again be a non-zero factor.

Combining all four terms of (31), (30) is rewritten as

32 $$\begin{aligned} \tilde{C}[k] = \sum _{u=1}^M \sum _{v=1}^M T_1[u,v] e^{j(\omega _u + \omega _v)k} + \sum _{u=1}^M T_2[u] e^{j \omega _u k} + T_3 \end{aligned}$$

where $T_1 = t_{11} - t_{21} - t_{31} - t_{41}$, etc., and $T_2$, $T_3$ are non-zero only when $X_0 \ne 0$.

Note that $T_2$ will have $X_0$ (or $X_0^{\star }$) as a factor, whereas $T_3$ will involve higher power terms of $X_0$ (or $X_0^{\star }$). As a consequence, when $X_0$ is small, as will be the case here, $T_3$ can be dropped from (32) retaining $T_2$ for small value (Sircar et al. 2015)). Rewriting (32) for small $X_0$, one obtains

33 $$\begin{aligned} \tilde{C}[k] = \sum _{u=1}^M \sum _{v=1}^M T_1[u,v] e^{j(\omega _u + \omega _v)k} + \sum _{u=1}^M T_2[u] e^{j \omega _u k} \end{aligned}$$

Note that even if $T_3$ is not negligible, the mode corresponding to the dropped term from (32) is real unity, which can be easily identified and discarded.

Comparing (13) and (33), it can be observed that the $\tilde{C}$-sequence consists of the square and product modes of the signal, together with the low amplitude original signal modes. If there are *M* modes in the sampled signal, the number of modes in the $\tilde{C}$-sequence will be $L = M + M(M+1)/2 = M(M+3)/2 \,\,$. Consequently, the sequence will satisfy the linear prediction equations of order more than *L*. Remember that the unity mode may also be present.

In the complex FM sinusoidal signal case, the $\tilde{C}$-sequence will have the form

34 $$\begin{aligned}\tilde{C}[k]&= \sum _{u=1}^M \sum _{v=1}^M\Bigg \{ T_1[u,v] e^{j(\omega _u+\omega _v)k} + T_2[u,v] e^{j(\omega _u+\omega _v+\xi _u)k} \nonumber \\ & \quad + T_3[u,v] e^{j(\omega _u+\omega _v-\xi _u)k} + T_4[u,v] e^{j(\omega _u+\omega _v+\xi _v)k} + T_5[u,v] e^{j(\omega _u+\omega _v-\xi _v)k} \Bigg \} \nonumber \\ &\quad + \sum _{u=1}^M \Bigg \{ T_6[u]e^{j \omega _u k} + T_7[u]e^{j( \omega _u + \xi _u) k} + T_8[u]e^{j( \omega _u - \xi _u) k} \Bigg \} \end{aligned}$$

under the assumption that the signal *X*[*n*] comprises of narrow-band FM sinusoids with small values of $\beta _u$’s. Note that $T_6$, $T_7$, $T_8$ are non-zero only when $X_0 \ne 0$.

In the complex linear chirp signal case, the $\tilde{C}$-sequence will have the form

35 $$\begin{aligned} \tilde{C}[k] = \sum _{u=1}^M \sum _{v=1}^M T_1[u,v] e^{j((\omega _u+\omega _v)k+(\gamma _u+\gamma _v){k^2}/2)} + \sum _{u=1}^M T_2[u]e^{j(\omega _u k+\gamma _u {k^2}/2)} \end{aligned}$$

under the assumption that the chirp rates are comparable, i.e., $(\gamma _u-\gamma _v)$ is very small. Note that $T_2$ is non-zero only when $X_0 \ne 0$.

In the presence of additive noise, the $\tilde{C}$-sequence may deviate, but it is likely that this deviation will be small when the superimposed noise is zero-mean Gaussian and uncorrelated with the signal. Remember that we are doing time averaging here.

## Simulation study

Simulation study is carried out for the complex sinusoidal signals, complex FM sinusoidal signals, and complex linear chirp signals. The common simulation parameters used for all the signals are the number of realizations equal to 500, the multiplicative noise amplitude $\alpha _i$ to be i.i.d. and Rician distributed, and its phase $\phi _i$ to be i.i.d. and $U[0,2\pi )$, and the additive noise *W*[*n*] to be complex zero-mean white circular Gaussian process.

### Complex sinusoidal signals

The signal *Y*[*n*] taken for simulation consists of *M* complex sinusoidal signals in multiplicative and additive noise.

36 $$\begin{aligned} Y[n]=\sum \limits _{i=1}^{M}\alpha _{i}e^{\jmath (\omega _{i}n+\phi _{i})}+W[n] \end{aligned}$$

where *M* = 2, the angular frequencies $\omega _{i}$ = $2\pi \left( {f_{i}}/{f_s}\right)$ with $f_{1}=70$ Hz and $f_{2}=150$ Hz, the sampling rate $f_s=800$ Hz, and the number of data points $N=513$. The amplitude $\alpha _{i}$ and the phase $\phi _{i}$ of the multiplicative noise and the additive noise *W*[*n*] are as stated above.

The sequence $\bar{Y}[n]$ is computed by subtracting the mean of *Y*[*n*] from each value of the data sequence. The new sequence $\bar{Y}[n]$ is used to compute the FOTC as given by (30).

The resulting FOTC $\tilde{C}[k]$, being the sum of *L* complex sinusoids, satisfies the *L*th order prediction equation. The order *L* becomes $L=M(M+3)/2=5$. We use the extended order modelling for noise immunity and form forward prediction error filter (PEF) $\mathcal{{D}}_J(z)$ as

37 $$\begin{aligned} \mathcal{{D}}_J(z)&= \mathcal{{A}}_L(z) \mathcal{{B}}_{J-L}(z) \\&=\prod _{i=1}^L \left( 1-z^{-1}z_i \right) \left[ 1+b_1z^{-1}+ \cdots +b_{J-L}z^{-(J-L)} \right] \nonumber \\&=1+d_1z^{-1}+d_2z^{-2}+ \cdots +d_Jz^{-J} \nonumber \end{aligned}$$

for an arbitrary $(J-L)$ degree polynomial $\mathcal{{B}}_{J-L}(z)$, $J>L$. The corresponding linear prediction equation in the $\tilde{C}$-values can be written as

38 $$\begin{aligned} \tilde{C}[k]=-\sum \limits _{m=1}^{J}d_{m}\tilde{C}[k-m], \qquad \text {for} -K+J \le k \le K \end{aligned}$$

where the extended model order $J=10$, $d_{i}$’s are the prediction coefficients, and the sequence $\tilde{C}[k]$ is available for $\left\{ k=-K,\ldots ,0,\ldots ,K\right\}$.

We can write (38) in matrix form as,

39 $$\begin{aligned} \mathbf{Cd=0} \end{aligned}$$

where $$[\mathbf{C}]_{{\ell }m} = \tilde{C}[-K+J+\ell -m]$$; $$\ell = 0,1,\ldots ,2K-J$$; $$m = 0,1,\ldots ,J$$, and

$$\mathbf{d} = [1\,\,\,d_1\,\,\,d_2\, \ldots \,d_J]^T$$.

**Figure 1:**
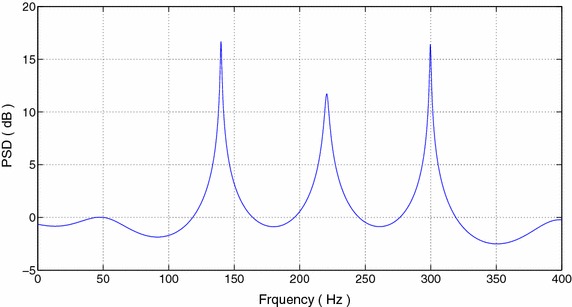
PSD of FOTC of complex sinusoidal signal.

**Figure 2:**
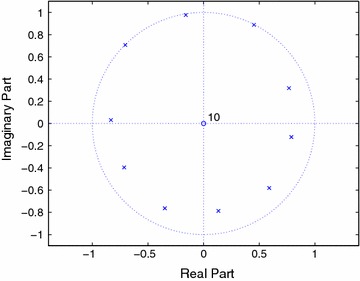
Pole-zero plots of FOTC with PEF order 10.

Once the prediction coefficient vector is known, we can calculate the power spectral density (PSD) as

40 $$\begin{aligned} S_{\tilde{C}}(f)=\frac{\sigma ^ {2} }{\left| D(f)\right| ^2} \end{aligned}$$

where $D(f)={\mathcal{{D}}_J}\left( e^{j2\pi f/f_s}\right)$. The computed PSD is shown in Figure 1 with $\sigma ^2=1$, and the pole-zero plot is shown in Figure 2. It can be seen that the noise poles are lying away from the unit circle, whereas the signal poles are located on the unit circle.

For $M>1$, the signal-to-noise ratio (SNR) in all the models is defined as

41 $$\begin{aligned} \text {SNR}=\frac{ \mathcal{{E}} \left\{ {\left| \sum \limits _{i=1}^{M}A_{i}\right| }^2 \right\} }{ \mathcal{{E}} \left\{ \left| W[n] \right| ^2\right\} } =\frac{ \sum \limits _{i=1}^{M}\sum \limits _{\substack{j=1 \\ j\ne i}}^{M}(\sigma _{A_i}^2 + \mu _{A_i}^{2}+ \mu _{A_i} \mu _{A_j})}{\sigma _W^2} \end{aligned}$$

where $\mu$ denotes the mean and $\sigma ^2$ stands for the variance.

**Figure 3:**
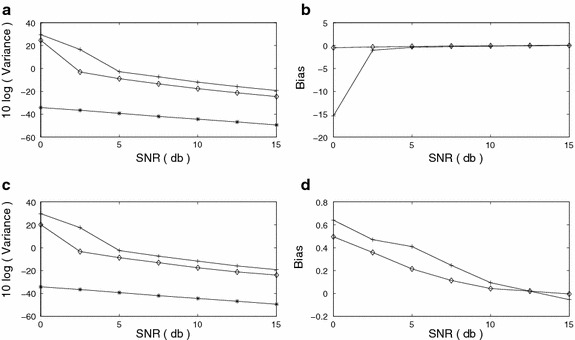
Sinusoidal frequency estimation (FOTC* diamond*, FOC* plus*, CRB* star*) **a** Variance, *f*_1_ **b** Bias, *f*_1_ **c** Variance, *f*_2_ **d** Bias, *f*_2_.

We compare our results with the results obtained by the method developed in (Swami 1994). The FOC values defined in (Swami 1994) are used to get the alternative set of estimates, whereas the proposed method uses the FOTC values defined in (30). The bias and variance versus SNR plots for $f_1$ and $f_2$ are shown in Figure 3a–d. The CR bound is also shown for comparison with the variance plot. The rate of decay of variance in each of the methods is similar to that of the CR bound. The variance computed for the proposed method is closer to the CR bound than the variance computed for the method described in (Swami 1994). The bias of $f_1$ at SNR = 0 dB for the method of (Swami 1994) is large indicating that the method is inaccurate at this noise level. It is clearly visible in both the bias and variance plots that the method proposed in this paper performs better than the method of (Swami 1994) at all SNR levels.

### Complex FM sinusoidal signals

The complex FM sinusoidal signal *Y*[*n*] taken for simulation is

42 $$\begin{aligned} Y[n]=\sum _{i=1}^M \alpha _i e^{j\left[ \omega _i n+\beta _i \sin \left( \xi _in\right) +\phi _i\right] }+W[n] \end{aligned}$$

where $M=2$, the carrier angular frequencies $\omega _i=2\pi \left( {f_{c,i}}/{f_s}\right)$ with $f_{c,1}=180$ Hz and $f_{c,2}=80$ Hz, the modulating angular frequencies $\xi _i=2\pi \left( {f_{m,i}}/{f_s}\right)$ with $f_{m,1}=20$ Hz and $f_{m,2}=15$ Hz, the modulation indices $\beta _1=\beta _2=0.25$, $f_s=1000$ Hz, $N=513$, and $\alpha _i$, $\phi _i$, and *W*[*n*] are same as stated earlier.

The sequence $\bar{Y}[n]$ is computed by subtracting the mean of *Y*[*n*] from each value of the data sequence. The new sequence $\bar{Y}[n]$ is used to compute the FOTC as given by (30). The FM signal will contain modes corresponding to the carrier frequency $f_c$, and two side bands $f_c+f_m$ and $f_c-f_m$, and consequently, the resulting signal will have 6 modes. Thus, the FOTC will contain $L=M(M+3)/2=27$ modes.

**Figure 4:**
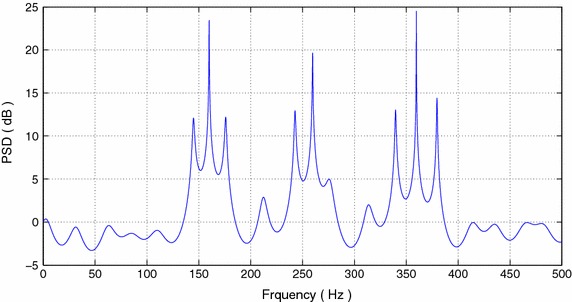
PSD of FOTC of complex FM signal.

**Figure 5:**
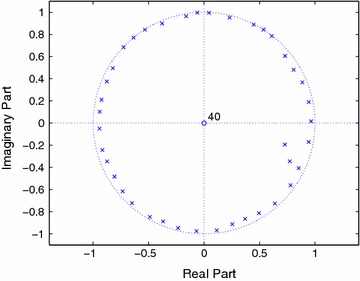
Pole-Zero plot of FOTC with PEF order 40.

**Figure 6:**
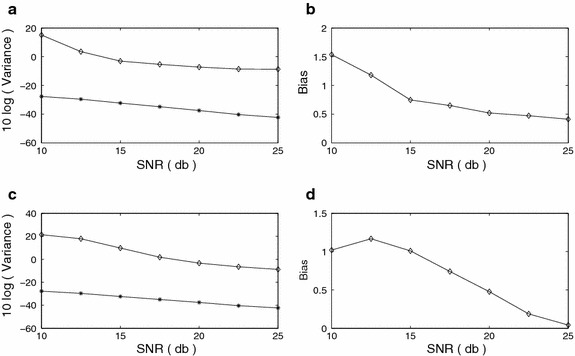
FM signal modulating frequency estimation (FOTC* diamond*, CRB* star*) **a** Variance, *f*_*m*,1_ **b** Bias, *f*_*m*,1_ **c** Variance, *f*_*m*,2_ **d** Bias, *f*_*m*,2_.

**Figure 7:**
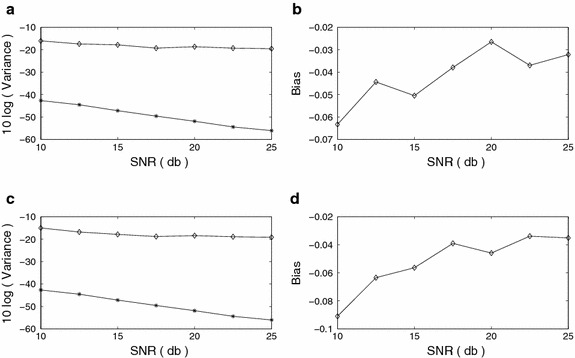
FM signal carrier frequency estimation (FOTC* diamond*, CRB* star*) **a** Variance, *f*_*c*,1_ **b** Bias, *f*_*c*,1_ **c** Variance, *f*_*c*,2_ **d** Bias, *f*_*c*,2_.

We use the extended model order $J=40$ to form the PEF, and the prediction coefficients are computed. The PSD computed using (40) is shown in Figure 4. The three clusters are centered at $2f_{c,1}$, $2f_{c,2}$, and $f_{c,1}+f_{c,2}$. The pole-zero plot is shown in Figure 5. It can be seen that the noise poles are lying away from the unit circle, whereas the signal poles are located on the unit circle. Figure 6a–d and 7a–d show the bias and variance versus SNR plots of estimation of modulating and carrier frequencies. The variance of estimate is compared with the CR bound.

Note that the variance versus SNR plots for $f_{m,1}$ and $f_{m,2}$ decay in the same rate as that of the corresponding CR bounds in Figure 6. The maximum bias for $f_{m,1}$ is 7.5 percent and that for $f_{m,2}$ is 8 percent in the range of SNR = [10, 25] dB. Below SNR = 10 dB, the bias for $f_{m,1}$ or $f_{m,2}$ is large, which indicates that the estimation is inaccurate below this SNR.

In Figure 7, we observe that the variance versus SNR plots for $f_{c,1}$ and $f_{c,2}$ do not follow the same rate of decay as that of the corresponding CR bounds. Note that the frequency estimation here is done with 27 modes, which lead to an ill-conditioned problem (Sircar and Sarkar 1988). In this case, the accuracy of estimation depends on both of the noise level and the conditioning of the estimation procedure at the particular noise level. The bias of $f_{c,1}$ or $f_{c,2}$ is found to be very small.

### Complex linear chirp signals

The complex linear chirp signal taken for simulation is

43 $$\begin{aligned} Y[n]=\sum _{i=1}^M \alpha _i e^{j(\omega _{i} n+\frac{\gamma _{i}}{2} n^{2}+ \phi _{i})}+W[n] \end{aligned}$$

where $M=2$, the on-set angular frequencies $\omega _i=2\pi \left( {f_{o,i}}/{f_s}\right)$ with $f_{o,1}=50$ Hz and $f_{o,2}=130$ Hz, the chirp rates $\gamma _i=2\pi \left( {f_{r,i}}/{f_s^2}\right)$ with $\;f_{r,1}=15$ and $\;f_{r,2}=30$, $f_s=800$ Hz, $N=1025$, and $\alpha _i$, $\phi _i$, and *W*[*n*] are same as stated earlier.

**Figure 8:**
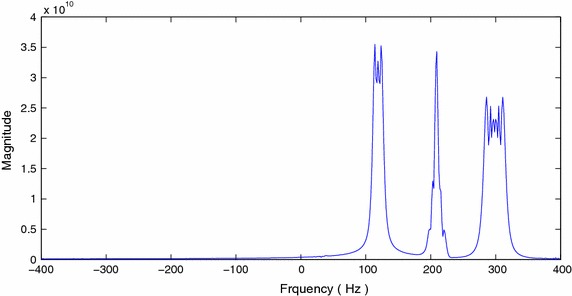
Magnitude spectrum of computed FOTC of chirp signal.

The sequence $\bar{Y}[n]$ is computed by subtracting the mean of *Y*[*n*] from each value of the data sequence. The new sequence $\bar{Y}[n]$ is used to compute the FOTC as given by (30). The magnitude spectrum of the computed FOTC is shown in Figure 8.

We compute the discrete ambiguity function (DAF) of the FOTC as given by (Peleg and Porat 1991)

44 $$\begin{aligned} \mbox{DAF} (\omega ,\ell )=\sum _{k=1}^{N-\ell }\tilde{C}[k+\ell ]\tilde{C}^{\star }[k]e^{-j\omega k} \end{aligned}$$

We use the lag parameter $\ell =(N-1)/2$ and compute the DAF of the FOTC. For $M=2$, when the mean of the finite data record is set to zero as discussed in "Deterministic signal case", the $\tilde{C}$-sequence will contain the following terms (35)

45 $$\begin{aligned} \tilde{C}[k]=T_{11}e^{j(2\omega _1 k+ \gamma _1 k^2)} + T_{12}e^{j(2\omega _2 k+ \gamma _2 k^2)} + T_{13}e^{j((\omega _1+\omega _2)k+(\gamma _1+\gamma _2){k^2}/2)} \end{aligned}$$

for negligible $T_2$ terms. Using (45) in (44), we get

46 $$\begin{aligned} {\rm DAF} (\omega ,\ell )&=\sum _{k=1}^{N-\ell }\left[ \sum _{i=1}^{6}B_{0,i}e^{j(2\omega _{d,i} k+ \gamma _{d,i} k^2)} \right. \nonumber \\ &\quad\left. + B_1 e^{j(2\gamma _1\ell )k} + B_2 e^{j(2\gamma _2\ell )k} + B_3 e^{j((\gamma _1+\gamma _2)\ell )k}\right] e^{-j\omega k} \end{aligned}$$

where $B_{0,i}$ and $B_m$, $m=1,2,3$ are the complex coefficients. The DAF expression of (46) contains 6 complex chirps and 3 complex sinusoids in frequency domain. The chirps are

$$\omega _{d,1}=(2(\omega _1-\omega _2)+2\gamma _1\ell )$$ with $$\gamma _{d,1}=((\gamma _1-\gamma _2)\ell )$$,

$$\omega _{d,2}=((\omega _1-\omega _2)+(\gamma _1+\gamma _2)\ell )$$ with $$\gamma _{d,2}=((\gamma _1-\gamma _2)\ell /2)$$,

$$\omega _{d,3}=((\omega _1-\omega _2)+2\gamma _1\ell )$$ with $$\gamma _{d,3}=((\gamma _1-\gamma _2)\ell /2)$$,

$$\omega _{d,4}=((\omega _2-\omega _1)+(\gamma _1+\gamma _2)\ell )$$ with $$\gamma _{d,4}=((\gamma _1-\gamma _2)\ell /2)$$,

$$\omega _{d,5}=((\omega _2-\omega _1)+2\gamma _1\ell )$$ with $$\gamma _{d,5}=((\gamma _2-\gamma _1)\ell /2)$$, and

$$\omega _{d,6}=(2(\omega _2-\omega _1)+2\gamma _2\ell )$$ with $$\gamma _{d,6}=((\gamma _2-\gamma _1)\ell )$$.

**Figure 9:**
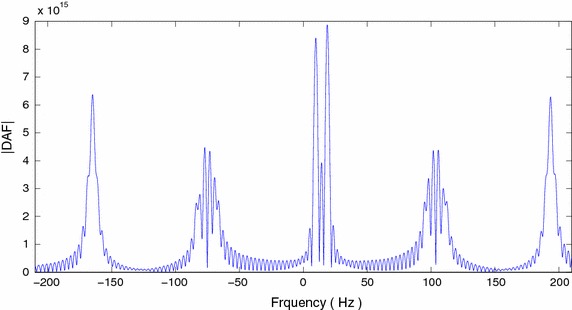
Discrete ambiguity function of FOTC of chirp signal.

The cross-terms due to multiple chirps can be attenuated/ eliminated by using the product high-order ambiguity function (Peleg and Porat 1991). The high-order ambiguity function of the FOTC is shown in Figure 9.

**Figure 10:**
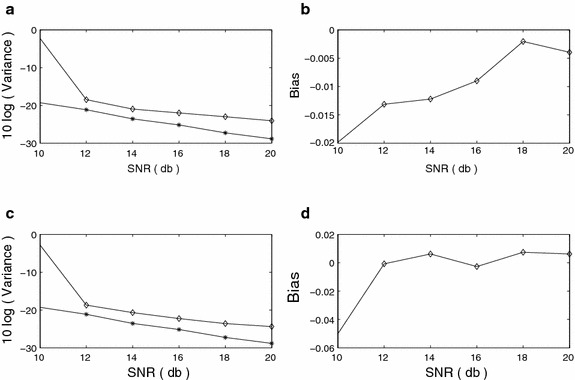
Chirp rate estimation (FOTC* diamond*, CRB* star*) **a** Variance, *f*_*r*,1_ **b** Bias, *f*_*r*,1_ **c** Variance, *f*_*r*,2_ **d** Bias, *f*_*r*,2_.

To compute the chirp rates, we find the peaks at $2\gamma _1 \ell$, $2\gamma _2 \ell$, and $(\gamma _1+\gamma _2)\ell$. In Figure 9, the three peaks near origin correspond to these frequencies. Since lag $\ell$ is known, the chirp rates can be estimated by detecting the above peaks. Once the chirp rates are known, by de-chirping the $\tilde{C}$-sequence, other parameters of chirps can be found (Peleg and Porat 1991; Barbarossa et al. 1998). Here, we show the results of estimation of the chirp rates. The bias and variance versus SNR plots of the chirp rates are shown in Figure 10a–d. The CR bound plots are shown together with the variance plots.

The plots show that the estimates of chirp rates are quite accurate for the SNR level above 12 dB. The variance of estimate is 3–5 dB higher than the CR bound in each case. The bias for $f_{r,1}$ or $f_{r,2}$ is very small. Thus, the parameters of the chirp signals in presence of additive and multiplicative noise can be estimated accurately by using the FOTC values of the signal and the method described in (Peleg and Porat 1991; Barbarossa et al. 1998).

## Conclusion

In this paper, the parameter estimation approach based on the symmetric fourth-order cumulant (FOC) or accumulated FOC (AFOC) is proposed for some stationary or non-stationary signals in multiplicative and additive noise. The derivations of the symmetric FOC are carried out for the multi-component complex sinusoidal, complex FM sinusoidal and complex linear chirp signals.

In case of parameter estimation of complex sinusoidal signal, the proposed method performs better than the method presented in (Swami 1994) at all SNR levels, even though the latter is also another method based on the fourth order statistics.

The simulation results show that using the method based on the new definition of the FOC or AFOC as developed in this paper, the parameters of various stationary and non-stationary signals can be estimated accurately in multiplicative and additive noise environment. The CR bounds are computed in each case for comparison of the variances of estimated parameters.

The new definition of symmetric fourth-order moment and cumulant, as proposed in (Sircar et al. (2015)) and in this paper, reduces the dimension of fourth-order moment/cumulant drastically from three lag-variables to one lag-variable. Moreover, the symmetric FOC is found to be time-independent for some non-stationary signals like complex exponentials and linear chirps. In our future research, we like to explore the full potential of symmetric FOC by applying the proposed method for analysis of various other stationary and non-stationary signals in multiplicative and additive noise. As further research, we need to present results for comparison of performance of our method and that of the methods based on the NLLS and cyclic statistics.

## Appendix A: The CR bound for complex sinusoids

The signal *Y*[*n*] comprising of *M* complex sinusoidal signals in multiplicative and additive noise is given as

47 $$\begin{aligned} Y[n]=\sum \limits _{i=1}^{M}A_{i}[n]e^{\jmath \omega _{i}n}+W[n],\quad n=0,\ldots ,N-1 \end{aligned}$$

where $A_{i}[n]$ are the multiplicative noise processes and *W*[*n*] is the additive noise process.

The Cramer-Rao bound (CRB) for a single complex sinusoid in multiplicative complex-valued circularly symmetric Gaussian noise and independent circular complex white Gaussian additive noise is considered in (Ghogho et al. 2001). Here, we consider the multi-component signals with multiplicative and additive noise. The noise processes are the complex-valued Gaussian processes with the following properties. We also examine the random variable case (4). Our assumptions about the noise processes are given below:

1. $A_{i}[n]$ are the complex-valued Gaussian processes, circularly symmetric around their mean $\mu _{i} e^{\jmath \psi _{i}}$, $\mu _{i}\ge 0$, $-\pi \le \psi _{i} < \pi$, i.e., $\mathbf a _{i}\sim CN(\mu _{i} e^{\jmath \psi _{i}}\mathbf 1 ,\sigma _{A_{i}}^2 \mathbf I )$, where $\mathbf a _{i}$ is a $(N\times 1)$ vector generated from $A_{i}[n]$. Moreover, $A_{i}[n]$ are i.i.d. and independent of additive noise.
2. *W*[*n*] is a zero-mean circular complex white Gaussian noise process, i.e., $\mathbf w \sim CN(\mathbf{{0}},\sigma _{W}^2 \mathbf I )$, where $\mathbf w$ is a $(N\times 1)$ vector generated from *W*[*n*].

We can write (47) as

48 $$\begin{aligned} \mathbf y =\sum \limits _{i=1}^{M}\mathbf E _{i}\mathbf a _{i}+\mathbf w \end{aligned}$$

where $\mathbf E _{i}= {\rm Diag} \left[ e^{\jmath \omega _{i}n}; n=0,\ldots ,N-1\right]$, and $\mathbf y$ is a vector of size $(N\times 1)$ generated from *Y*[*n*].

The mean vector is given by

49 $$\begin{aligned} \mathbf {m}_{Y}={\mathcal {E}}\{\mathbf {y}\}=\sum \limits _{i=1}^{M}\mu _{i} e^{\jmath \psi _{i}} \mathbf {E}_{i}\mathbf {1} \end{aligned}$$

where $\mathcal{{E}}$ is the expectation operator and **1** is a vector of ones of size $(N\times 1)$. Let $\bar{\mathbf{y }}=\mathbf y -\mathbf m _{Y}$; then $\bar{\mathbf{y }}$ will be circularly symmetric because of the assumption of circular symmetry and mutual independence of $A_{i}[n]$’s and *W*[*n*] (Ghogho et al. 2001). The statistics of $\bar{\mathbf{y }}$ can be described by only the correlation matrix $\mathbf R _{Y}=\mathcal{{E}}\{\bar{\mathbf{y }}\bar{\mathbf{y }}^{H}\}$, and the psuedo-correlation matrix $\mathbf U _{Y}=\mathcal{{E}}\{\bar{\mathbf{y }}\bar{\mathbf{y }}^{T}\}$ will be zero. We can write

50 $$\begin{aligned} \mathbf R _{Y}=\mathcal{{E}}\{\bar{\mathbf{y }}\bar{\mathbf{y }}^{H}\} =\sum \limits _{i=1}^{M}\mathbf E _{i}\mathbf R _{A_i}\mathbf E _{i}^{H}+\sigma _{W}^{2} \mathbf I \end{aligned}$$

where **I** is the identity matrix of size $(N\times N)$ and $\mathcal{{E}} \left\{ \bar{\mathbf{a _i}}\bar{\mathbf{a _i}}^H \right\} =\mathbf R _{A_i}$.

For the complex Gaussian probability density function (PDF), the Fisher information matrix (FIM) is given by (Kay 2010)

51 $$\begin{aligned} \mathbf J _{\theta _{i},\theta _{l}}={\rm tr} \left\{ \mathbf R _{Y}^{-1}\frac{\partial \mathbf R _{Y}}{\partial \theta _{i}}\mathbf R _{Y}^{-1}\frac{\partial \mathbf R _{Y}}{\partial \theta _{l}} \right\} +2{\rm Re} \left\{ \frac{\partial \mathbf m _{Y}^H}{\partial \theta _{i}}\mathbf R _{Y}^{-1}\frac{\partial \mathbf m _{Y}}{\partial \theta _{l}}\right\} . \end{aligned}$$

Let the parameter vector be $\Theta =[\omega _{1}\; \omega _{2} \;...\; \omega _{M}]$. Consider the *i*th term of $\mathbf R _{Y}$ in (50), $\mathbf R _{Y_i}=\mathbf E _{i}\mathbf R _{A_i}\mathbf E _{i}^{H}+\sigma _{W}^{2} \mathbf I$. Since the process $A_i[n]$ is i.i.d., the derivative of $\mathbf R _{Y}$ with respect to any of the defined parameters will be zero. So only the mean vector will contribute to the FIM, and the first term of (51) will be zero. The partial derivatives of the mean vector will be

52 $$\begin{aligned} \frac{\partial \mathbf m _{Y}^H}{\partial \omega _{i}}= \frac{\partial }{\partial \omega _{i}}\left\{ \sum \limits _{i=1}^{M}\mu _{i} e^{-\jmath \psi _{i}} \mathbf 1 ^{T}\mathbf E _i^{H}\right\} =-\jmath \mu _i e^{-\jmath \psi _i} \mathbf 1 ^{T}\mathbf D \mathbf E _{i}^{H} \end{aligned}$$

where $$\mathbf D = {\rm Diag} \left[ 0,\ldots ,N-1\right]$$, and

53 $$\begin{aligned} \frac{\partial \mathbf m _{Y}}{\partial \omega _{i}}= \frac{\partial }{\partial \omega _{i}}\left\{ \sum \limits _{i=1}^{M}\mu _{i} e^{\jmath \psi _{i}} \mathbf E _i \mathbf 1 \right\} =\jmath \mu _i e^{\jmath \psi _i} \mathbf D \mathbf E _{i}\mathbf 1 \end{aligned}$$

On substitution of the computed values, (51) gives the FIM entries $J_{\theta _{i},\theta _{l}}$. The entries are given as

54 $$\begin{aligned} J_{\omega _{i}\omega _{i}}= \mu _i^2 \,2{\rm Re} \left\{ \mathbf 1 ^{T}\mathbf R _{Y}^{-1}\mathbf D ^{2}\mathbf 1 \right\} \end{aligned}$$

and

55 $$\begin{aligned} J_{\omega _{i}\omega _{l}}= \mu _i\mu _l \,2{\rm Re} \left\{ e^{\jmath (\psi _l-\psi _i)} \mathbf 1 ^{T}\mathbf R _{Y}^{-1}\mathbf D ^{2}\mathbf E _{i}^{H}\mathbf E _{l}\mathbf 1 \right\} \end{aligned}$$

where the computed derivatives of the mean are substituted.

The CR bounds are given by the diagonal elements of the inverse of FIM, $\mathbf J ^{-1}$, and these are evaluated at the true value of the parameters, i.e.,

56 $$\begin{aligned} {\rm CRB} (\omega _{i})=\left[ \mathbf J ^{-1}\right] _{i,i} \end{aligned}$$

Now consider the signal given in (47). When $A_{i}[n]=A_{i}$ is the circularly symmetric complex Gaussian random variable, (47) reduces to

57 $$\begin{aligned} Y[n]=\sum \limits _{i=1}^{M}A_{i}e^{\jmath \omega _{i}n}+W[n],\quad n=0,\ldots ,N-1 \end{aligned}$$

where $$A_{i}$$ = $$\alpha _{i}e^{\jmath \phi _{i}}$$, and the amplitude $$\alpha _{i}$$ is the Rayleigh/ Rician random variable, the phase $$\phi _{i}$$$$\sim U[0,2\pi )$$. Let us consider the non-zero mean case, and assume that $$A_{i}$$ is circularly symmetric around mean $$\mu _{i} e^{\jmath \psi _{i}}$$. Note that the mean vector of *Y*[*n*] will be same as (49) and using similar arguments as above the correlation matrix can be shown to be

58 $$\begin{aligned} \mathbf R _{Y}=\mathcal{{E}}\{\bar{\mathbf{y }}\bar{\mathbf{y }}^{H}\} =\sum \limits _{i=1}^{M}\mathbf E _{i}\sigma _{A_i}^2\mathbf 1 \mathbf 1 ^{T}\mathbf E _{i}^{H}+\sigma _{W}^{2} \mathbf I \end{aligned}$$

where $\mathcal{{E}} \left\{ \bar{\mathbf{a}}_i\bar{\mathbf{a}}_i^H \right\} =\sigma _{A_i}^2\mathbf 1 \mathbf 1 ^{T}$, which is independent of the frequencies to be estimated. So the resulting CR bound expressions will be similar to random process case with $\mathbf R _{A_i}=\sigma _{A_i}^2\mathbf 1 \mathbf 1 ^{T}$. The CR bound expressions for random variable case can be obtained in a straightforward way by evaluating the partial derivatives given in (51).

## Appendix B: The CR bound for complex FM sinusoids

Consider the sum of complex FM sinusoidal signals in multiplicative and additive noise

59 $$\begin{aligned} Y[n]=\sum \limits _{i=1}^{M}A_{i}[n]e^{\jmath [\omega _{i}n+\beta _{i} \sin (\xi _{i}n)]}+W[n], \quad n=0,...,N-1 \end{aligned}$$

where the assumptions related to the multiplicative and additive noise are same as in Appendix A. The above equation can be written as

60 $$\begin{aligned} \mathbf y =\sum \limits _{i=1}^{M}\mathbf E _{i}\mathbf a _{i}+\mathbf w \end{aligned}$$

where $\mathbf E _{i}= {\rm Diag} \left[ e^{\jmath [\omega _{i}n+\beta _i \sin (\xi _{i}n)]}; n=0,\ldots ,N-1\right]$, and $\mathbf y$, $\mathbf a _{i}$, $\mathbf w$ are vectors of size $(N\times 1)$.

Following the similar procedure and same assumptions as in appendix A, we get the mean vector

61 $$\begin{aligned} \mathbf {m}_{Y}={\mathcal {E}}\{\mathbf {y}\}=\sum \limits _{i=1}^{M}\mu _{i} e^{\jmath \psi _{i}} \mathbf {E}_{i}\mathbf {1} \end{aligned}$$

where **1** is a vector of ones of size $(N\times 1)$ and the correlation matrix is

62 $$\begin{aligned} \mathbf R _{Y}=\mathcal{{E}}\{\bar{\mathbf{y }}\bar{\mathbf{y }}^{H}\} =\sum \limits _{i=1}^{M}\mathbf E _{i}\mathbf R _{A_i}\mathbf E _{i}^{H}+\sigma _{W}^{2} \mathbf I \end{aligned}$$

where $\mathbf I$ is the identity matrix of size $(N\times N)$ and $\mathcal{{E}} \left\{ \bar{\mathbf{a _i}}\bar{\mathbf{a _i}}^H \right\} =\mathbf R _{A_i}$.

Consider the *i*th term of $\mathbf R _{Y}$ in (62), $\mathbf R _{Y_i}=\mathbf E _{i}\mathbf R _{A_i}\mathbf E _{i}^{H}+\sigma _{W}^{2} \mathbf I$. Since the random process $A_i[n]$ is i.i.d., the derivative of $\mathbf R _{Y}$ with respect to any of the defined parameters will be zero. So only the mean vector will contribute to the FIM.

Let the parameter vector be $\Theta =[ \xi _{1} \;\omega _{1}\; \xi _{2} \;\omega _{2}\;\cdots \; \xi _{M}\; \omega _{M}]$. The partial derivatives of the mean vector are

63 $$\begin{aligned} \frac{\partial \mathbf m _{Y}^H}{\partial \xi _{i}}= \frac{\partial }{\partial \xi _{i}}\left\{ \sum \limits _{i=1}^{M}\mu _i e^{\jmath \psi _i}\mathbf 1 ^\mathbf{T }\mathbf E _i^{H}\right\} =-\jmath \mu _i e^{-\jmath \psi _i}\beta _i \mathbf 1 ^\mathbf{T }\mathbf D \mathbf C _{i}\mathbf E _{i}^{H}, \end{aligned}$$

64 $$\begin{aligned} \frac{\partial \mathbf m _{Y}^H}{\partial \omega _{i}}= \frac{\partial }{\partial \omega _{i}}\left\{ \sum \limits _{i=1}^{M}\mu _i e^{\jmath \psi _i}\mathbf 1 ^\mathbf{T }\mathbf E _i^{H}\right\} =-\jmath \mu _i e^{-\jmath \psi _i} \mathbf 1 ^\mathbf{T }\mathbf D \mathbf E _{i}^{H} \end{aligned}$$

where $$\mathbf C _{i}= {\rm Diag} \left[ \cos (\xi _{i}n); n=0,\ldots ,N-1\right]$$, $$\mathbf D = {\rm Diag} \left[ 0,\ldots ,N-1\right]$$, and

65 $$\begin{aligned} \frac{\partial \mathbf m _{Y}}{\partial \xi _{i}}= \frac{\partial }{\partial \xi _{i}}\left\{ \sum \limits _{i=1}^{M}\mu _{i} e^{\jmath \psi _{i}} \mathbf E _{i}\mathbf 1 \right\} =\jmath \mu _i e^{\jmath \psi _i}\beta _i \mathbf D \mathbf C _{i}\mathbf E _{i}\mathbf 1 , \end{aligned}$$

66 $$\begin{aligned} \frac{\partial \mathbf m _{Y}}{\partial \omega _{i}}= \frac{\partial }{\partial \omega _{i}}\left\{ \sum \limits _{i=1}^{M}\mu _{i} e^{\jmath \psi _{i}} \mathbf E _{i}\mathbf 1 \right\} =\jmath \mu _i e^{\jmath \psi _i} \mathbf D \mathbf E _{i}\mathbf 1 . \end{aligned}$$

On substitution of the computed values, (51) gives the FIM entries $J_{\theta _{i},\theta _{l}}$. The entries are

67 $$\begin{aligned} J_{\xi _{i}\xi _{i}}= 2\mu _i^2\beta _i^2\,{\rm Re} \left\{ \mathbf 1 ^{T}\mathbf R _{Y}^{-1}\mathbf D ^{2}\mathbf C _{i}^{2}\mathbf 1 \right\} \end{aligned}$$

68 $$\begin{aligned} J_{\xi _{i}\xi _{l}}= 2\mu _i\mu _l\beta _i\beta _l\,{\rm Re} \left\{ e^{\jmath (\psi _l-\psi _i)} \mathbf 1 ^{T}\mathbf R _{Y}^{-1}\mathbf D ^{2}\mathbf C _{i}\mathbf C _{l}\mathbf E _{i}^{H}\mathbf E _{l}\mathbf 1 \ \right\} \end{aligned}$$

69 $$\begin{aligned} J_{\xi _{i}\omega _{i}}= 2\mu _i^2\beta _i\,{\rm Re} \left\{ \mathbf 1 ^{T}\mathbf R _{Y}^{-1}\mathbf D ^{2}\mathbf C _{i}\mathbf 1 \ \right\} \end{aligned}$$

70 $$\begin{aligned} J_{\xi _{i}\omega _{l}}= 2\mu _i\mu _l\beta _i\,{\rm Re} \left\{ e^{\jmath (\psi _l-\psi _i)} \mathbf 1 ^{T}\mathbf R _{Y}^{-1}\mathbf D ^{2}\mathbf C _{i}\mathbf E _{i}^{H}\mathbf E _{l}\mathbf 1 \ \right\} \end{aligned}$$

71 $$\begin{aligned} J_{\omega _{i}\xi _{i}}= 2\mu _i^2\beta _i\,{\rm Re} \left\{ \mathbf 1 ^{T}\mathbf R _{Y}^{-1}\mathbf D ^{2}\mathbf C _{i}\mathbf 1 \right\} \end{aligned}$$

72 $$\begin{aligned} J_{\omega _{i}\xi _{l}}= 2\mu _i\mu _l\beta _l\,{\rm Re} \left\{ e^{\jmath (\psi _l-\psi _i)} \mathbf 1 ^{T}\mathbf R _{Y}^{-1}\mathbf D ^{2}\mathbf C _{l}\mathbf E _{i}^{H}\mathbf E _{l}\mathbf 1 \ \right\} \end{aligned}$$

73 $$\begin{aligned} J_{\omega _{i}\omega _{i}}= 2\mu _i^2\,{\rm Re} \left\{ \mathbf 1 ^{T}\mathbf R _{Y}^{-1}\mathbf D ^{2}\mathbf 1 \ \right\} \end{aligned}$$

74 $$\begin{aligned} J_{\omega _{i}\omega _{l}}= 2\mu _i\mu _l\,{\rm Re} \left\{ e^{\jmath (\psi _l-\psi _i)} \mathbf 1 ^{T}\mathbf R _{Y}^{-1}\mathbf D ^{2}\mathbf E _{i}^{H}\mathbf E _{l}\mathbf 1 \ \right\} \end{aligned}$$

where the computed derivatives of the mean are substituted.

The CR bounds are given by the diagonal elements of the inverse of FIM, $\mathbf J ^{-1}$, and these are evaluated at the true value of the parameters, i.e.,

75 $$\begin{aligned} {\rm CRB} (\xi _{i})=\left[ \mathbf J ^{-1}\right] _{2(i-1)+1,2(i-1)+1},\, {\rm CRB} (\omega _{i})=\left[ \mathbf J ^{-1}\right] _{2(i-1)+2,2(i-1)+2}. \end{aligned}$$

Now consider the signal given in (59). When $A_{i}[n]=A_{i}$ is the circularly symmetric complex Gaussian random variable, (59) reduces to

76 $$\begin{aligned} Y[n]=\sum \limits _{i=1}^{M}A_{i}e^{\jmath (\omega _{i}n+\beta _{i} \sin (\xi _{i}n))}+W[n],\quad n=0,\ldots,N-1 \end{aligned}$$

where $$A_{i}$$= $$\alpha _{i}e^{\jmath \phi _{i}}$$, and the magnitude $$\alpha _{i}$$ is the Rayleigh/ Rician random variable, the phase $$\phi _{i}$$$$\sim U[0,2\pi )$$. Let us consider the non-zero mean case and assume that $$A_{i}$$ is circularly symmetric around mean $$\mu _{i} e^{\jmath \psi _{i}}$$. Note that the mean vector of *Y*[*n*] will be same as (61), and using similar arguments as before the correlation matrix can be shown to be

77 $$\begin{aligned} \mathbf R _{Y}=\mathcal{{E}}\{\bar{\mathbf{y }}\bar{\mathbf{y }}^{H}\} =\sum \limits _{i=1}^{M}\mathbf E _{i}\sigma _{A_i}^2\mathbf 1 \mathbf 1 ^{T}\mathbf E _{i}^{H}+\sigma _{W}^{2} \mathbf I \end{aligned}$$

where $\mathcal{{E}} \left\{ \bar{\mathbf{a}}_i\bar{\mathbf{a}}_i^H \right\} =\sigma _{A_i}^2\mathbf 1 \mathbf 1 ^{T}$ which is independent of the frequencies to be estimated. So the resulting CR bound expressions will be similar to the random process case with $\mathbf R _{A_i}=\sigma _{A_i}^2\mathbf 1 \mathbf 1 ^{T}$. The CR bound expressions for the random variable case can be obtained in a straightforward way by evaluating the partial derivatives given in (51).

## Appendix C: The CR bound for complex linear chirps

Consider the sum of complex linear chirp signals in multiplicative and additive noise

78 $$\begin{aligned} Y[n]=\sum _{i=1}^M A_{i}[n] e^{j(\omega _{i}n+\gamma _{i}(n)^{2}/2)}+W(n), \quad n=0,\ldots ,N-1 \end{aligned}$$

where the assumptions related to the multiplicative and additive noise are same as in appendix A. The above equation can be written as

79 $$\begin{aligned} \mathbf y =\sum \limits _{i=1}^{M}\mathbf E _{i}\mathbf a _{i}+\mathbf w \end{aligned}$$

where $\mathbf E _{i}= {\rm Diag} \left[ e^{\jmath (\omega _{i}n+\gamma _{i}(n)^{2}/2)}; n=0,\ldots ,N-1\right]$, and $\mathbf y$, $\mathbf a _{i}$, $\mathbf w$ are vectors of size $(N\times 1)$.

The mean vector is given by

80 $$\begin{aligned} \mathbf {m}_{Y}={\mathcal {E}}\{\mathbf {y}\}=\sum \limits _{i=1}^{M}\mu _{i} e^{\jmath \psi _{i}} \mathbf {E}_{i}\mathbf {1} \end{aligned}$$

where **1** is a vector of ones of size $(N\times 1)$, and the correlation matrix

81 $$\begin{aligned} \mathbf R _{Y}=\mathcal{{E}}\{\bar{\mathbf{y }}\bar{\mathbf{y }}^{H}\} =\sum \limits _{i=1}^{M}\mathbf E _{i}\mathbf R _{A_i}\mathbf E _{i}^{H}+\sigma _{W}^{2} \mathbf I \end{aligned}$$

where $\mathbf I$ is the identity matrix of size $(N\times N)$ and $\mathcal{{E}} \left\{ \bar{\mathbf{a }}\bar{\mathbf{a }}^H \right\} =\mathbf R _{A_i}$.

Let the parameter vector be $\Theta =[\gamma _{1}\; \omega _{1}\; \gamma _{2} \; \omega _2\; ...\; \gamma _{M}\; \omega _{M}]$. The derivative of $\mathbf R _{Y}$ with respect to any of the defined parameters will be zero. So only the mean vector will contribute to the FIM. The partial derivatives of the mean vector will be

82 $$\begin{aligned} \frac{\partial \mathbf m _{Y}^H}{\partial \gamma _{i}}= \frac{\partial }{\partial \gamma _{i}}\left\{ \sum \limits _{i=1}^{M}\mu _{i} e^{-\jmath \psi _{i}} \mathbf 1 ^{T}\mathbf E _i^{H}\right\} =-\jmath \mu _i e^{-\jmath \psi _i}\frac{1}{2} \mathbf 1 ^{T}\mathbf D ^2\mathbf E _{i}^{H} \end{aligned}$$

83 $$\begin{aligned} \frac{\partial \mathbf m _{Y}^H}{\partial \omega _{i}}= \frac{\partial }{\partial \omega _{i}}\left\{ \sum \limits _{i=1}^{M}\mu _{i} e^{-\jmath \psi _{i}} \mathbf 1 ^{T}\mathbf E _i^{H}\right\} =-\jmath \mu _i e^{-\jmath \psi _i} \mathbf 1 ^{T}\mathbf D \mathbf E _{i}^{H} \end{aligned}$$

where $$\mathbf D = {\rm Diag} \left[ 0,\ldots ,N-1\right]$$, and

84 $$\begin{aligned} \frac{\partial \mathbf m _{Y}}{\partial \gamma _{i}}= \frac{\partial }{\partial \gamma _{i}}\left\{ \sum \limits _{i=1}^{M}\mu _{i} e^{\jmath \psi _{i}} \mathbf E _i \mathbf 1 \right\} =\jmath \mu _i e^{\jmath \psi _i} \frac{1}{2} \mathbf D ^2\mathbf E _{i}\mathbf 1 \end{aligned}$$

85 $$\begin{aligned} \frac{\partial \mathbf m _{Y}}{\partial \omega _{i}}= \frac{\partial }{\partial \omega _{i}}\left\{ \sum \limits _{i=1}^{M}\mu _{i} e^{\jmath \psi _{i}} \mathbf E _i \mathbf 1 \right\} =\jmath \mu _i e^{\jmath \psi _i} \mathbf D \mathbf E _{i}\mathbf 1 \end{aligned}$$

On substitution of the computed values, (51) gives the FIM entries $J_{\theta _{i},\theta _{l}}$. The entries are given as

86 $$\begin{aligned} J_{\gamma _{i}\gamma _{i}}= \mu _i^2 \,\frac{1}{2}{\rm Re} \left\{ \mathbf 1 ^{T}\mathbf R _{Y}^{-1}\mathbf D ^{4}\mathbf 1 \right\} \end{aligned}$$

87 $$\begin{aligned} J_{\gamma _{i} \omega _i}= \mu _i^2 \,{\rm Re} \left\{ \mathbf 1 ^{T}\mathbf R _{Y}^{-1}\mathbf D ^{3}\mathbf 1 \right\} \end{aligned}$$

88 $$\begin{aligned} J_{\gamma _{i}\gamma _{l}}= \mu _i\mu _l \,\frac{1}{2}{\rm Re} \left\{ e^{\jmath (\psi _l-\psi _i)} \mathbf 1 ^{T}\mathbf R _{Y}^{-1}\mathbf D ^{4}\mathbf E _{i}^{H}\mathbf E _{l}\mathbf 1 \right\} \end{aligned}$$

89 $$\begin{aligned} J_{\gamma _{i}\omega _{l}}= \mu _i\mu _l \, {\rm Re} \left\{ e^{\jmath (\psi _l-\psi _i)} \mathbf 1 ^{T}\mathbf R _{Y}^{-1}\mathbf D ^{3}\mathbf E _{i}^{H}\mathbf E _{l}\mathbf 1 \right\} \end{aligned}$$

90 $$\begin{aligned} J_{\omega _{i} \gamma _{i}}=\mu _i^2 \,{\rm Re} \left\{ \mathbf 1 ^{T}\mathbf R _{Y}^{-1}\mathbf D ^{3}\mathbf 1 \right\} \end{aligned}$$

91 $$\begin{aligned} J_{\omega _{i}\omega _{i}}= 2\mu _i^2 {\rm Re} \left\{ \mathbf 1 ^{T}\mathbf R _{Y}^{-1}\mathbf D ^{2}\mathbf 1 \right\} \end{aligned}$$

92 $$\begin{aligned} J_{\omega _{i} \gamma _{l}}= \mu _i\mu _l \, {\rm Re} \left\{ e^{\jmath (\psi _l-\psi _i)} \mathbf 1 ^{T}\mathbf R _{Y}^{-1}\mathbf D ^{3}\mathbf E _{i}^{H}\mathbf E _{l}\mathbf 1 \right\} \end{aligned}$$

93 $$\begin{aligned} J_{\omega _{i} \omega _{l}}= \mu _i\mu _l \,2 {\rm Re} \left\{ e^{\jmath (\psi _l-\psi _i)} \mathbf 1 ^{T}\mathbf R _{Y}^{-1}\mathbf D ^{2}\mathbf E _{i}^{H}\mathbf E _{l}\mathbf 1 \right\} \end{aligned}$$

where the computed derivatives of the mean are substituted.

The CR bounds are given by the diagonal elements of the inverse of FIM, $\mathbf J ^{-1}$, and these are evaluated at the true value of the parameters, i.e.,

94 $$\begin{aligned} {\rm CRB} (\gamma _{i})=\left[ \mathbf J ^{-1}\right] _{2(i-1)+1,2(i-1)+1},\, {\rm CRB} (\omega _{i})=\left[ \mathbf J ^{-1}\right] _{2(i-1)+2,2(i-1)+2}. \end{aligned}$$

For the random variable $A_i$ case, we can show that the mean vector will be same as (80) and the correlation matrix will be

95 $$\begin{aligned} \mathbf R _{Y}=\mathcal{{E}}\{\bar{\mathbf{y }}\bar{\mathbf{y }}^{H}\} =\sum \limits _{i=1}^{M}\mathbf E _{i}\sigma _{A_i}^2\mathbf 1 \mathbf 1 ^{T}\mathbf E _{i}^{H}+\sigma _{W}^{2} \mathbf I \end{aligned}$$

where $\mathcal{{E}} \left\{ \bar{\mathbf{a}}_i\bar{\mathbf{a}}_i^H \right\} =\sigma _{A_i}^2\mathbf 1 \mathbf 1 ^{T}$ which will be independent of the parameters to be estimated. So the resulting CR bound expressions will be similar to the random process case with $\mathbf R _{A_i}=\sigma _{A_i}^2\mathbf 1 \mathbf 1 ^{T}$. The CR bound expressions for the random variable case can be obtained in a straightforward way by evaluating the partial derivatives given in (51).
